# 1-Phenyl-2-*p*-tolyl-1*H*-benzimidazole

**DOI:** 10.1107/S160053681300264X

**Published:** 2013-02-02

**Authors:** T. Mohandas, K. Jayamoorthy, P. Sakthivel, J. Jayabharathi

**Affiliations:** a Shri Angalamman College of Engineering and Technology, Siruganoor, Trichirappalli, Tamilnadu 621 105, India; b Annamalai University, Chidambaram, Tamilnadu, India; c Department of Physics, Urumu Dhanalakshmi college, Trichirappalli, Tamilnadu 620 019, India

## Abstract

In the title compound, C_20_H_16_N_2_, the benzimidazole ring system forms dihedral angles of 28.50 (7) and 72.44 (7)° with the tolyl and phenyl rings, respectively. In the crystal, mol­ecules are linked into chains along the *a-*axis direction by weak C—H⋯N inter­actions. The crystal structure also features C—H⋯π inter­actions.

## Related literature

For applications of benzimidazole derivatives, see: Fang *et al.* (2007 ▶); Ge *et al.* (2008 ▶); Lai *et al.* (2008 ▶); Shin *et al.* (2007 ▶). For their biological activity, see: Garuti *et al.* (1999 ▶); Matsuno *et al.* (2000 ▶) and for their therapeutic applications, see: Can-Eke *et al.* (1998 ▶); Richter (1997 ▶). For standard bond lengths, see: Allen *et al.* (1987 ▶).
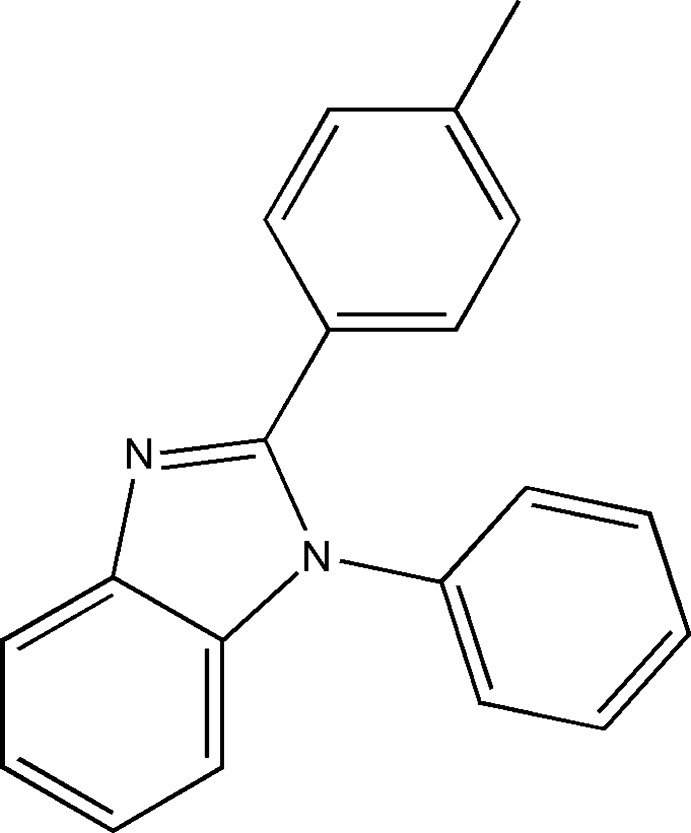


## Experimental

### 

#### Crystal data

C_20_H_16_N_2_
*M* *_r_* = 284.35
Orthorhombic, 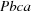

*a* = 15.6755 (4) Å
*b* = 9.3509 (6) Å
*c* = 21.1976 (8) Å
*V* = 3107.1 (2) Å^3^
*Z* = 8
Mo *K*α radiation
μ = 0.07 mm^−1^
*T* = 293 K
0.30 × 0.30 × 0.25 mm

#### Data collection

Bruker Kappa APEXII diffractometer
Absorption correction: multi-scan (*SADABS*; Bruker, 2008 ▶) *T*_min_ = 0.966, *T*_max_ = 0.997
15284 measured reflections
2523 independent reflections
1840 reflections with *I* > 2σ(*I*)
*R*_int_ = 0.034

#### Refinement

*R*[*F* ^2^ > 2σ(*F* ^2^)] = 0.036
*wR*(*F* ^2^) = 0.103
*S* = 1.01
2523 reflections
201 parameters
H-atom parameters constrained
Δρ_max_ = 0.12 e Å^−3^
Δρ_min_ = −0.11 e Å^−3^

### 

Data collection: *APEX2* (Bruker, 2008 ▶); cell refinement: *SAINT* (Bruker, 2008 ▶); data reduction: *SAINT*; program(s) used to solve structure: *SHELXS97* (Sheldrick, 2008 ▶); program(s) used to refine structure: *SHELXL97* (Sheldrick, 2008 ▶); molecular graphics: *PLATON* (Spek, 2009 ▶); software used to prepare material for publication: *PLATON*.

## Supplementary Material

Crystal structure: contains datablock(s) global, I. DOI: 10.1107/S160053681300264X/fy2081sup1.cif

Structure factors: contains datablock(s) I. DOI: 10.1107/S160053681300264X/fy2081Isup2.hkl

Supplementary material file. DOI: 10.1107/S160053681300264X/fy2081Isup3.cml

Additional supplementary materials:  crystallographic information; 3D view; checkCIF report

## Figures and Tables

**Table 1:** Hydrogen-bond geometry (Å, °) *Cg*1 is the centroid of the C15–C20 phenyl ring.

*D*—H⋯*A*	*D*—H	H⋯*A*	*D*⋯*A*	*D*—H⋯*A*
C16—H16⋯N1^i^	0.93	2.50	3.337 (2)	149
C20—H20⋯*Cg*1^ii^	0.93	2.80	3.707 (2)	166

Symmetry codes: (i) 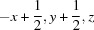; (ii) 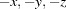.

**Fig. 1.**
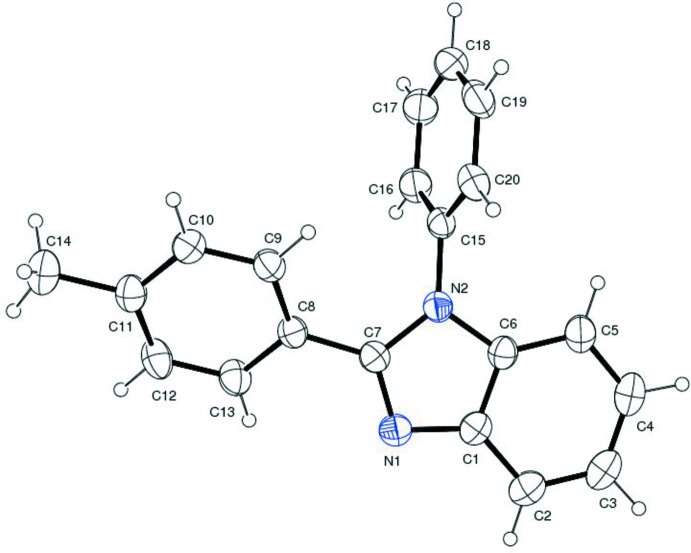
The molecular structure and labelling scheme for (I) with displacement ellipsoids for non-H atoms drawn at the 30% probability level.

**Fig. 2.**
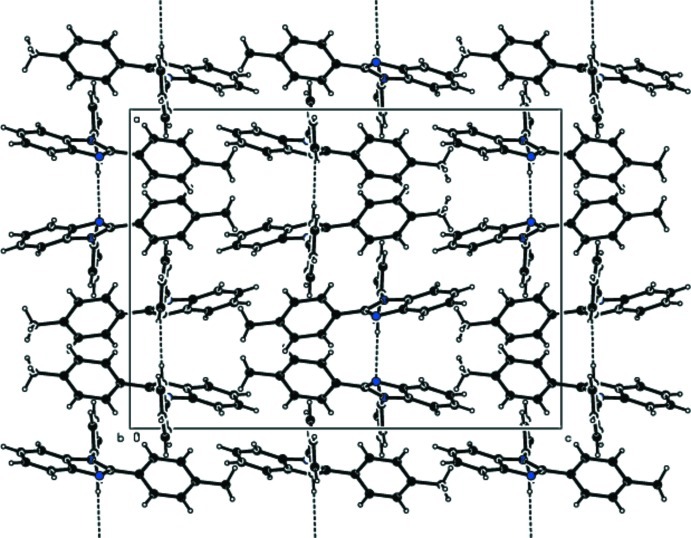
Packing diagram of (I). Dashed lines indicate intermolecular hydrogen bonding interactions.

## supplementary crystallographic information

### Comment

Benzimidazoles play an important role in the development of coordination chemistry. Benzimidazole derivatives have attracted considerable attention because of their biological and pharmaceutical activities (Garuti *et al.*, 1999; Matsuno *et al.*, 2000).

Substituted benzimidazole derivatives have diverse therapeutic applications, as they exibit antiulcerative (Richter, 1997) and antioxidant (Can-Eke *et al.*, 1998) activities.

Because of their wide optical absorption, bright luminescence and bipolar transport characteristics, fused imidazole derivatives, such as benzoimidazoles and phenanthroimidazoles (Fang *et al.*, 2007; Ge *et al.*, 2008; Lai *et al.*, 2008), have been used in the fabrication of light-emitting devices, employing them as electron-transporting layer and as sensitizers in dye-sensitized solar cells (Shin *et al.*, 2007). Benzimidazole derivatives possess antioxidant, antimicrobial and antifungal activities. In view of their importance, the structure determination of the title compound was carried out and the results are presented herein.

The asymmetric unit contains one molecuale of the title compound (I). The bond lengths and bond angles are within normal ranges (Allen *et al.*, 1987). X-ray analysis confirms the molecular structure and atom connectivity of the compound as illustrated in Fig. 1.

The planar benzimidazole ring system N1/C7/N2/C6/C1–C5 is oriented with respect to the adjacent tolyl and phenyl rings, C8–C14 and C15–C20, with dihedral angles of 28.50 (7)° and 72.44 (7)° respectively. In the crystal structure, the molecules are linked into chains along the *a* axis by intermolecular C—H···N hydrogen bonds. The crystal structure is further stabilized by the weak intermolecular C—H···π(*Cg*1) interaction (Table 1), where *Cg*1 is the centre of gravity of the C15–C20 phenyl ring.

The packing view of the title compound is shown in Fig 2.

### Experimental

*N*-phenyl-*o*-phenylenediamine (17 mmol, 3.128 g) was dissolved in ethanol (10 ml), then 4-methyl benzaldehyde (17 mmol, 2.1 ml) and ammonium acetate (3 g) were added, maintaining the temperature at 80°C for about an hour. The reaction mixture was refluxed for 48 h and the completion of the reaction was monitored by TLC. After completion of the reaction, the mixture was extracted with dichloromethane. The solid separated was purified by column chromatography using petroleum ether as the eluent. Yield: 2.82 g (50%). Single crystals were grown in ethanol as a solvent at room temperature.

### Refinement

The hydrogen atoms were placed in calculated positions with C—H= 0.93 Å to 0.96 Å, and refined in the riding model with fixed isotropic displacement parameters: *U*_iso_(H) = 1.5*U*_eq_(C) for methyl group and *U*_iso_(H) = 1.2*U*_eq_(C) for other H atoms. The methyl H atoms of tolyl group are allowed to rotate (AFIX 137) to optimal position.

### Crystal data

**Table:**

C_20_H_16_N_2_	*F*(000) = 1200
*M**_r_* = 284.35	*D*_x_ = 1.216 Mg m^−^^3^
Orthorhombic, *P**b**c**a*	Mo *K*α radiation, λ = 0.71073 Å
Hall symbol: -P 2ac 2ab	Cell parameters from 3879 reflections
*a* = 15.6755 (4) Å	θ = 2.7–22.5°
*b* = 9.3509 (6) Å	µ = 0.07 mm^−^^1^
*c* = 21.1976 (8) Å	*T* = 293 K
*V* = 3107.1 (2) Å^3^	Block, colourless
*Z* = 8	0.30 × 0.30 × 0.25 mm

### Data collection

**Table:**

Bruker Kappa APEXII diffractometer	2523 independent reflections
Radiation source: rotating anode	1840 reflections with *I* > 2σ(*I*)
Graphite monochromator	*R*_int_ = 0.034
Detector resolution: 18.4 pixels mm^-1^	θ_max_ = 24.3°, θ_min_ = 2.3°
ω and φ scan	*h* = −18→13
Absorption correction: multi-scan (*SADABS*; Bruker, 2008)	*k* = −8→10
*T*_min_ = 0.966, *T*_max_ = 0.997	*l* = −24→18
15284 measured reflections	

### Refinement

**Table:**

Refinement on *F*^2^	Secondary atom site location: difference Fourier map
Least-squares matrix: full	Hydrogen site location: inferred from neighbouring sites
*R*[*F*^2^ > 2σ(*F*^2^)] = 0.036	H-atom parameters constrained
*wR*(*F*^2^) = 0.103	*w* = 1/[σ^2^(*F*_o_^2^) + (0.0474*P*)^2^ + 0.5482*P*] where *P* = (*F*_o_^2^ + 2*F*_c_^2^)/3
*S* = 1.01	(Δ/σ)_max_ < 0.001
2523 reflections	Δρ_max_ = 0.12 e Å^−^^3^
201 parameters	Δρ_min_ = −0.11 e Å^−^^3^
0 restraints	Extinction correction: *SHELXL97* (Sheldrick, 2008), Fc^*^=kFc[1+0.001xFc^2^λ^3^/sin(2θ)]^-1/4^
Primary atom site location: structure-invariant direct methods	Extinction coefficient: 0.0073 (6)

### Special details

**Table:**

Geometry. Bond distances, angles *etc*. have been calculated using the rounded fractional coordinates. All su's are estimated from the variances of the (full) variance-covariance matrix. The cell e.s.d.'s are taken into account in the estimation of distances, angles and torsion angles
Refinement. Refinement of *F*^2^ against ALL reflections. The weighted *R*-factor *wR* and goodness of fit *S* are based on *F*^2^, conventional *R*-factors *R* are based on *F*, with *F* set to zero for negative *F*^2^. The threshold expression of *F*^2^ > σ(*F*^2^) is used only for calculating *R*-factors(gt) *etc*. and is not relevant to the choice of reflections for refinement. *R*-factors based on *F*^2^ are statistically about twice as large as those based on *F*, and *R*- factors based on ALL data will be even larger.

### Fractional atomic coordinates and isotropic or equivalent isotropic displacement parameters (Å^2^)

**Table:**

	*x*	*y*	*z*	*U*_iso_*/*U*_eq_	
N1	0.14753 (9)	−0.13901 (15)	0.07038 (6)	0.0552 (5)	
N2	0.09742 (9)	0.08161 (13)	0.08884 (6)	0.0487 (5)	
C1	0.12646 (10)	−0.12606 (18)	0.13367 (8)	0.0531 (6)	
C2	0.13195 (12)	−0.2254 (2)	0.18248 (9)	0.0672 (7)	
C3	0.10524 (14)	−0.1835 (2)	0.24119 (9)	0.0743 (8)	
C4	0.07406 (13)	−0.0470 (2)	0.25226 (9)	0.0751 (8)	
C5	0.06882 (11)	0.0531 (2)	0.20511 (8)	0.0641 (7)	
C6	0.09523 (10)	0.01008 (17)	0.14593 (7)	0.0507 (6)	
C7	0.12994 (9)	−0.01370 (17)	0.04511 (7)	0.0472 (6)	
C8	0.14252 (10)	0.01833 (17)	−0.02203 (7)	0.0470 (5)	
C9	0.09213 (11)	0.11433 (17)	−0.05513 (8)	0.0536 (6)	
C10	0.10375 (12)	0.13481 (18)	−0.11886 (8)	0.0569 (6)	
C11	0.16546 (11)	0.06158 (19)	−0.15234 (8)	0.0562 (6)	
C12	0.21529 (11)	−0.0342 (2)	−0.11910 (8)	0.0647 (7)	
C13	0.20433 (11)	−0.05620 (19)	−0.05528 (8)	0.0597 (7)	
C14	0.17583 (14)	0.0840 (2)	−0.22227 (9)	0.0782 (8)	
C15	0.07647 (10)	0.22994 (17)	0.08275 (7)	0.0473 (6)	
C16	0.14063 (13)	0.32822 (19)	0.07561 (8)	0.0615 (7)	
C17	0.12051 (17)	0.4708 (2)	0.07146 (9)	0.0791 (9)	
C18	0.03838 (19)	0.5144 (2)	0.07484 (9)	0.0793 (9)	
C19	−0.02647 (15)	0.4162 (2)	0.08192 (8)	0.0741 (8)	
C20	−0.00744 (12)	0.2715 (2)	0.08613 (7)	0.0586 (7)	
H2	0.15302	−0.31698	0.17546	0.0806*	
H3	0.10809	−0.24820	0.27444	0.0892*	
H4	0.05623	−0.02266	0.29271	0.0901*	
H5	0.04856	0.14504	0.21259	0.0769*	
H9	0.04999	0.16546	−0.03403	0.0642*	
H10	0.06909	0.19980	−0.13997	0.0683*	
H12	0.25734	−0.08529	−0.14034	0.0777*	
H13	0.23879	−0.12174	−0.03432	0.0717*	
H14A	0.12372	0.05913	−0.24338	0.1173*	
H14B	0.22126	0.02453	−0.23762	0.1173*	
H14C	0.18915	0.18246	−0.23039	0.1173*	
H16	0.19721	0.29863	0.07359	0.0738*	
H17	0.16371	0.53792	0.06628	0.0949*	
H18	0.02558	0.61134	0.07237	0.0949*	
H19	−0.08286	0.44676	0.08386	0.0889*	
H20	−0.05058	0.20415	0.09114	0.0703*	

### Atomic displacement parameters (Å^2^)

**Table:**

	*U*^11^	*U*^22^	*U*^33^	*U*^12^	*U*^13^	*U*^23^
N1	0.0569 (9)	0.0543 (9)	0.0543 (9)	0.0053 (7)	−0.0008 (7)	0.0016 (7)
N2	0.0514 (8)	0.0480 (8)	0.0466 (8)	−0.0015 (6)	0.0053 (6)	−0.0017 (6)
C1	0.0496 (10)	0.0571 (11)	0.0526 (11)	−0.0049 (8)	−0.0022 (8)	0.0039 (8)
C2	0.0721 (13)	0.0649 (12)	0.0646 (12)	−0.0018 (10)	−0.0062 (10)	0.0113 (10)
C3	0.0794 (14)	0.0847 (15)	0.0589 (13)	−0.0093 (12)	−0.0015 (10)	0.0201 (11)
C4	0.0791 (14)	0.0967 (16)	0.0495 (11)	−0.0075 (12)	0.0088 (9)	0.0050 (11)
C5	0.0679 (13)	0.0721 (12)	0.0522 (11)	−0.0022 (10)	0.0083 (9)	−0.0026 (9)
C6	0.0488 (10)	0.0558 (10)	0.0475 (10)	−0.0053 (8)	0.0034 (8)	0.0010 (8)
C7	0.0415 (9)	0.0499 (10)	0.0501 (10)	−0.0007 (7)	0.0011 (7)	−0.0023 (8)
C8	0.0443 (9)	0.0509 (9)	0.0459 (9)	−0.0013 (7)	0.0003 (7)	−0.0038 (8)
C9	0.0544 (11)	0.0542 (10)	0.0521 (10)	0.0055 (8)	0.0036 (8)	−0.0039 (8)
C10	0.0615 (11)	0.0570 (10)	0.0522 (11)	0.0019 (9)	−0.0021 (8)	0.0026 (8)
C11	0.0537 (11)	0.0655 (11)	0.0494 (10)	−0.0088 (9)	0.0029 (8)	−0.0034 (9)
C12	0.0527 (11)	0.0859 (13)	0.0556 (12)	0.0088 (10)	0.0074 (8)	−0.0126 (10)
C13	0.0517 (11)	0.0727 (12)	0.0548 (11)	0.0128 (9)	−0.0003 (8)	−0.0027 (9)
C14	0.0827 (15)	0.0974 (15)	0.0545 (12)	−0.0074 (12)	0.0090 (10)	0.0015 (10)
C15	0.0540 (11)	0.0474 (10)	0.0404 (9)	−0.0020 (8)	0.0028 (7)	−0.0048 (7)
C16	0.0659 (12)	0.0582 (12)	0.0603 (11)	−0.0100 (9)	0.0070 (9)	−0.0054 (9)
C17	0.112 (2)	0.0552 (12)	0.0700 (13)	−0.0163 (12)	0.0153 (12)	−0.0064 (10)
C18	0.131 (2)	0.0530 (12)	0.0538 (12)	0.0133 (14)	0.0079 (12)	−0.0039 (9)
C19	0.0857 (15)	0.0854 (15)	0.0511 (12)	0.0331 (13)	0.0013 (10)	−0.0125 (10)
C20	0.0570 (12)	0.0664 (12)	0.0525 (10)	0.0050 (9)	0.0034 (8)	−0.0104 (9)

### Geometric parameters (Å, º)

**Table:**

N1—C1	1.387 (2)	C16—C17	1.373 (3)
N1—C7	1.318 (2)	C17—C18	1.352 (4)
N2—C6	1.383 (2)	C18—C19	1.378 (3)
N2—C7	1.383 (2)	C19—C20	1.388 (3)
N2—C15	1.431 (2)	C2—H2	0.9300
C1—C2	1.393 (3)	C3—H3	0.9300
C1—C6	1.389 (2)	C4—H4	0.9300
C2—C3	1.370 (3)	C5—H5	0.9300
C3—C4	1.387 (3)	C9—H9	0.9300
C4—C5	1.372 (3)	C10—H10	0.9300
C5—C6	1.381 (2)	C12—H12	0.9300
C7—C8	1.468 (2)	C13—H13	0.9300
C8—C9	1.386 (2)	C14—H14A	0.9600
C8—C13	1.386 (2)	C14—H14B	0.9600
C9—C10	1.377 (2)	C14—H14C	0.9600
C10—C11	1.381 (2)	C16—H16	0.9300
C11—C12	1.382 (2)	C17—H17	0.9300
C11—C14	1.506 (3)	C18—H18	0.9300
C12—C13	1.379 (2)	C19—H19	0.9300
C15—C16	1.371 (2)	C20—H20	0.9300
C15—C20	1.373 (2)		
			
C1—N1—C7	105.41 (13)	C15—C20—C19	118.57 (18)
C6—N2—C7	106.49 (12)	C1—C2—H2	121.00
C6—N2—C15	122.81 (13)	C3—C2—H2	121.00
C7—N2—C15	130.43 (13)	C2—C3—H3	119.00
N1—C1—C2	130.20 (16)	C4—C3—H3	119.00
N1—C1—C6	110.19 (14)	C3—C4—H4	119.00
C2—C1—C6	119.61 (16)	C5—C4—H4	119.00
C1—C2—C3	117.71 (17)	C4—C5—H5	122.00
C2—C3—C4	121.64 (18)	C6—C5—H5	122.00
C3—C4—C5	121.73 (18)	C8—C9—H9	120.00
C4—C5—C6	116.43 (17)	C10—C9—H9	120.00
N2—C6—C1	105.72 (13)	C9—C10—H10	119.00
N2—C6—C5	131.41 (15)	C11—C10—H10	119.00
C1—C6—C5	122.88 (15)	C11—C12—H12	119.00
N1—C7—N2	112.19 (13)	C13—C12—H12	119.00
N1—C7—C8	123.20 (14)	C8—C13—H13	120.00
N2—C7—C8	124.60 (14)	C12—C13—H13	120.00
C7—C8—C9	123.12 (14)	C11—C14—H14A	109.00
C7—C8—C13	118.97 (14)	C11—C14—H14B	109.00
C9—C8—C13	117.81 (15)	C11—C14—H14C	109.00
C8—C9—C10	120.76 (16)	H14A—C14—H14B	109.00
C9—C10—C11	121.86 (16)	H14A—C14—H14C	110.00
C10—C11—C12	117.08 (16)	H14B—C14—H14C	109.00
C10—C11—C14	120.82 (16)	C15—C16—H16	120.00
C12—C11—C14	122.09 (16)	C17—C16—H16	120.00
C11—C12—C13	121.77 (16)	C16—C17—H17	120.00
C8—C13—C12	120.72 (16)	C18—C17—H17	120.00
N2—C15—C16	119.43 (15)	C17—C18—H18	120.00
N2—C15—C20	119.29 (15)	C19—C18—H18	120.00
C16—C15—C20	121.25 (16)	C18—C19—H19	120.00
C15—C16—C17	119.32 (19)	C20—C19—H19	120.00
C16—C17—C18	120.5 (2)	C15—C20—H20	121.00
C17—C18—C19	120.47 (19)	C19—C20—H20	121.00
C18—C19—C20	119.9 (2)		
			
C7—N1—C1—C2	179.96 (18)	C4—C5—C6—C1	−0.6 (3)
C7—N1—C1—C6	0.33 (18)	C4—C5—C6—N2	179.05 (17)
C1—N1—C7—N2	−0.49 (17)	N2—C7—C8—C13	154.00 (15)
C1—N1—C7—C8	−179.56 (14)	N1—C7—C8—C9	149.15 (16)
C6—N2—C7—N1	0.47 (18)	N1—C7—C8—C13	−27.1 (2)
C15—N2—C7—N1	174.56 (15)	N2—C7—C8—C9	−29.8 (2)
C6—N2—C15—C16	103.65 (18)	C13—C8—C9—C10	−0.3 (2)
C7—N2—C15—C16	−69.6 (2)	C7—C8—C13—C12	176.83 (16)
C6—N2—C15—C20	−74.4 (2)	C7—C8—C9—C10	−176.52 (16)
C7—N2—C15—C20	112.39 (18)	C9—C8—C13—C12	0.4 (3)
C7—N2—C6—C1	−0.23 (17)	C8—C9—C10—C11	0.0 (3)
C15—N2—C6—C1	−174.88 (14)	C9—C10—C11—C12	0.2 (3)
C7—N2—C6—C5	−179.95 (17)	C9—C10—C11—C14	178.90 (17)
C15—N2—C6—C5	5.4 (3)	C14—C11—C12—C13	−178.73 (17)
C6—N2—C7—C8	179.52 (14)	C10—C11—C12—C13	−0.1 (3)
C15—N2—C7—C8	−6.4 (3)	C11—C12—C13—C8	−0.3 (3)
C6—C1—C2—C3	0.4 (3)	N2—C15—C16—C17	−178.28 (15)
N1—C1—C6—C5	179.69 (15)	C20—C15—C16—C17	−0.3 (2)
N1—C1—C6—N2	−0.06 (19)	N2—C15—C20—C19	178.20 (14)
N1—C1—C2—C3	−179.16 (18)	C16—C15—C20—C19	0.2 (2)
C2—C1—C6—C5	0.0 (3)	C15—C16—C17—C18	0.5 (3)
C2—C1—C6—N2	−179.73 (15)	C16—C17—C18—C19	−0.6 (3)
C1—C2—C3—C4	−0.3 (3)	C17—C18—C19—C20	0.5 (3)
C2—C3—C4—C5	−0.4 (3)	C18—C19—C20—C15	−0.3 (2)
C3—C4—C5—C6	0.8 (3)		

### Hydrogen-bond geometry (Å, º)

Cg1 is the centroid of the C15–C20 phenyl ring.

**Table:**

*D*—H···*A*	*D*—H	H···*A*	*D*···*A*	*D*—H···*A*
C16—H16···N1^i^	0.9300	2.5000	3.337 (2)	149.00
C20—H20···*Cg*1^ii^	0.9300	2.7988	3.707 (2)	165.79

Symmetry codes: (i) −*x*+1/2, *y*+1/2, *z*; (ii) −*x*, −*y*, −*z*.
